# Evaluation of the effect of red cabbage waste on performance, egg quality, and yolk oxidative stability of laying Japanese quails (*Coturnix coturnix Japonica*)

**DOI:** 10.1007/s11250-025-04336-4

**Published:** 2025-02-27

**Authors:** Erinç Gümüş, Behlül Sevim, Selim Sırakaya, Canan İriş, Oğuzhan Kahraman, Ainhoa Sarmiento-García

**Affiliations:** 1 Department of Veterinary, Eskil Vocational School, Aksaray University, 68800 EskilAksaray, Türkiye; 2 Department of Food Processing, Technical Vocational School, Aksaray University, 68100 Aksaray, Türkiye; 3 Independent Researcher, 68800 EskilAksaray, Türkiye; 4 Department of Animal Nutrition and Nutritional Disorders, Faculty of Veterinary Medicine, Selcuk University, 42130 Konya, Türkiye; 5 Área de Producción Animal, Departamento de Construcción y Agronomía, Facultad de Ciencias Agrarias y Ambientales, Universidad de Salamanca, 37007 Salamanca, Spain

**Keywords:** Egg quality, Japanese quail, Oxidative stability, Performance, Red cabbage waste

## Abstract

This study investigates the effects of red cabbage waste (RCW) as a dietary supplement on the performance, egg quality, and oxidative stability of yolk in laying Japanese quails. Given its rich phenolic content, RCW has been investigated as a natural feed additive to reduce food waste and enhance poultry diets. In a six-week trial, 120 quails were divided into 4 groups with 5 replicates and fed diets with 0.0%, 0.5%, 1.0%, and 2.0% RCW. Performance parameters, internal and external egg quality, and antioxidant status were measured. RCW supplementation had no significant impact on overall quail performance. Quails fed with 0.5% RCW showed notable improvements in internal egg quality, especially in the albumen index, Haugh unit, and eggshell thickness. Additionally, incorporating 0.5% RCW into the diet improved the yolk’s antioxidant activity, as evidenced by reduced MDA levels. However, other egg quality traits, including the antioxidant capacity of the yolk, had deteriorated with concentrations above 0.5%, indicating that a moderate inclusion of 0.5% RCW is the most favourable option. The findings underscore that RCW could be sustainably used to improve egg quality without compromising performance, while managing food waste, presenting a potential circular economy solution. Nevertheless, further research is encouraged to optimize RCW levels and fully understand its benefits in poultry feed.

## Introduction

The management of food waste is a critical issue, with substantial implications for both Türkiye and the global community, leading to environmental challenges and substantial economic losses (Sarmiento-Garcia et al. 2023b, 2024). Estimates indicate that one-third of all food intended for human consumption is lost or wasted annually (Cakar et al. 2020; Sarmiento-García et al. 2024). In Türkiye, the annual total of biodegradable waste amounts to approximately 20 million metric tons, with over 8.6 million metric tons attributed to food waste alone (Salihoglu et al. 2018). Various strategies have been proposed to minimize or eliminate food waste. One potential solution is its inclusion in animal feed as a source of bioactive compounds, which can significantly influence animal nutrition (Salihoglu et al. 2018; Sarmiento-García et al. 2023b, 2024).

Improvements in both egg quality and laying production are crucial economic factors for table and hatching egg farms (Kamel et al. 2021). Recently, phytogenic feed additives have become a primary focus of research, largely due to their increasing favour as safer alternatives to synthetic additives and antibiotic growth promoters, which have been banned in the European Union for the past 20 years (Nunes et al. 2019; Ahmed et al. 2022). Natural ingredients, including fruits, seeds, flowers, and leaves from various plants containing phenolic compounds, can enhance animal performance by improving gastrointestinal morphology and egg quality, particularly by mitigating the adverse effects of lipid peroxidation on egg yolks (Ahmed et al. 2022; Nunes et al. 2019; Sarmiento-García et al. 2023a, 2024). Japanese quails hold significant value for avian research due to their small size, which makes them easy to handle and suitable for rearing in confined spaces (Sarmiento-García et al. 2023a). They are also known for many favorable attributes, such as their rapid reproductive cycles, strong resistance to numerous diseases, low production costs, and minimal initial investment (Kamel et al. 2021). These characteristics make them an excellent alternative for feed additive research on laying performance and egg quality traits.

Red cabbage (*Brassica oleracea* var. *capitata* f. *rubra*) is widely grown and noted for its vibrant colour and multiple health benefits (Ghareaghajlou et al. 2021). Cabbage varieties are rich in dietary fibre and essential minerals such as calcium, phosphorus, potassium, and vitamins A, K, and folate (Uuh‐Narvaez and Segura‐Campos 2021). Furthermore, red cabbage contains high levels of total phenolics and flavonoids. These phenolic compounds, primarily anthocyanins, mainly contribute to their antioxidant activity (Sarmiento-García et al. 2023b). Such compounds, including cyanidin glycosides, acetylated with various acids, contribute to red cabbage’s anti-inflammatory and antioxidant properties (Mizgier et al. 2016). Moreover, incorporating red cabbage into diets has been shown to benefit growth, obesity, and lipid profiles due to its phytochemical content and influence on various physiological processes (Ghareaghajlou et al. 2021).

Currently, over 30% of cabbage is discarded as waste within the food supply chain (Zhang et al. 2022). While reducing food waste remains a primary objective, reintegrating food by-products into the supply chain is essential for advancing the circular economy. In this context, plant-based by-products have been identified as valuable sources of bioactive compounds, comparable to those found in the original products (Gökmen et al. 2024; Sarmiento-García et al. 2024). Previous studies have suggested that incorporating various anthocyanin-rich plant by-products into livestock diets, such as red beet powder (Sarmiento-García et al. 2023a), bocaiuva pulp (Nunes et al. 2019), orange pulp (Goliomytis et al. 2018; Hussein et al. 2023), orange peel (Ahmed et al. 2022), dried olive pulp (Dedousi et al. 2022), or cassava residue (Almeida et al. 2020) could be a promising strategy for enhancing egg quality and production traits in both laying hens and quails.

Only one study has examined the effects of including dried cabbage leaves in laying hens’ diets, and no adverse effects on laying performance were observed with the supplementation of dried cabbage leaves (Mustafa and Baurhoo 2018). However, to our knowledge, only one study has been published on the effects of dried cabbage leaves on laying performance, egg quality red cabbage on laying performance, egg quality, and yolk oxidative stability in Japanese quails. Therefore, the authors hypothesize that including red cabbage waste (RCW) would positively impact performance, egg internal and external quality, and antioxidant parameters in the yolks of laying quails. Consequently, this study aimed to assess the potential use of RCW as a natural feed supplement in the diets of Japanese quails, focusing on its effects on performance, egg production parameters, internal and external egg quality traits, and yolk oxidative stability.

## Materials and the methods

All study protocols for the current research were approved by the Local Ethics Committee for Animal Experiments at the Faculty of Veterinary Medicine of Selçuk University and endorsed by an organized council (Decision No: 2023/134). Moreover, for the entirety of the trial, strict adherence to the guidelines established in the European Animal Protection Policy (EPCEU 2010) was adopted.

### Animals and feed materials

A total of 120 female 12-week-old Japanese quail (*Coturnix coturnix Japonica*), with a similar initial body weight (259.12 ± 11.91 g), were used in this study. The experiment was conducted at an indoor local farm in the Eskil District, Aksaray, Türkiye (latitude: 38°23′ 42′′, longitude: 33°25′68"). After arrival at the facilities, the quails were randomly allotted to 1 of 4 dietary treatments, each consisting of five replicates with six quails per replicate. Before the introduction of the animals, all housing units were thoroughly cleaned, well-ventilated, and prepared to ensure optimal conditions for the birds. The quails were housed in uniformly sized cages (45 cm wide × 30 long cm × 20 cm high), under the same environmental conditions and equipped with the necessary facilities for providing feed and water ad libitum. The experiment lasted over six weeks, from October to December 2023, and a 16-h daily lighting schedule was maintained throughout the experimental period.

Red cabbage waste (RCW) was sourced from a local market (Kaşınhan, Konya, Türkiye) where it was deemed unsuitable for human consumption. The fresh waste material was dried at 50 °C using a laboratory oven and subsequently ground to a fine powder with a particle size of 1 mm for use in the experimental diets. Before its inclusion, a proximate analysis was carried out following the procedures outlined by AOAC (2006): ash content was determined by incinerating the sample (method 942.05), protein content was measured using the Kjeldahl method (990.03), fat content was quantified with the Soxhlet extraction technique (2003.06), crude fibre was analysed according to the Weende method (978.10), and moisture content was established by drying at 105 °C (2001.12). Total polyphenol content was evaluated based on the methodology proposed by Mallek-Ayadi et al. (2017) and expressed as milligrams of gallic acid equivalents per 100 g of extract. The 1- diphenyl- 2- picrylhydrazyl (DPPH) radical scavenging activity was determined following the protocol described by Gökmen et al. (2024). The results are presented in Table 1.

**Table 1:** Chemical composition, total phenolic content, and reducing power of red cabbage powder

Parameters	Level
Metabolic Energy (kcal/kg)*	1270.31
Dry matter (%)	91.87
Crude protein (%)	17.65
Crude Fat (%)	1.56
Crude fiber (%)	13.44
Crude ash (%)	9.88
Strach (%)	6.23
DPPH (% reducing)**	84.63
Total phenolic content (g/kg GAE)	4081.71

*Metabolic Energy was calculated by formulation purposed by Carpenter and Clegs (1956)

**DPPH 2,2 diphenyl-1-picrylhydrazyl, *IC*_50_ The half maximal inhibitory concentration, *GAE* Gallic acid equivalent

The basal diet, without RCW supplementation, was formulated to fulfil the nutrient requirements for laying quails as outlined by the National Research Council (1994). The experimental diets were developed by incorporating RCW into the basal diet in mash form at inclusion levels of 0.5, 1.0, and 2.0%. RCW was considered as a feed additive rather than feedstuff. All diets were formulated to be isocaloric (2900 kcal ME/kg) and isonitrogenous (20% crude protein), ensuring no variations in energy or protein content among the experimental groups (Table 2). The chemical composition of the basal diet was analysed using the same methodologies applied to RCW (Table 2).

**Table 2:** Diet formulation and chemical composition of diets

Ingredients (%)	Experimental Diets (%)			
	**0**	**0.5**	**1.0**	**2.0**
Corn	52.80	52.54	52.27	51.74
Soybean meal (46% CP)	33.90	33.73	33.56	33.56
Sunflower oil	3.55	3.53	3.51	3.48
Limestone	7.55	7.51	7.47	7.40
Dicalcium phosphate	1.34	1.33	1.33	1.31
Common salt	0.40	0.40	0.40	0.39
Premix^1^	0.25	0.25	0.25	0.25
DL-methionine	0.21	0.21	0.21	0.21
Red Cabbage Waste	0.00	0.50	1.00	2.00
**Total**	**100.00**	**100.00**	**100.00**	**100.00**
**Chemical composition (%)**				
Metabolisable energy (kcal ME/kg)*	2918.96	2910.72	2902.51	2894.35
Dry matter	90.86	90.87	90.88	90.89
Crude protein	20.11	20.10	20.07	20.02
Crude ash	10.65	10.65	10.64	10.62
Crude fat	4.93	4.91	4.90	4.88
Crude fiber	4.13	4.18	4.22	4.27
Methionine + Cysteine	0.91	0.91	0.91	0.91
Calcium	3.22	3.20	3.19	3.17
Available phosphorus	0.38	0.38	0.38	0.37

^1^Per kg, Premix provides 20,000 IU vitamin A (trans retinol acetate), 0.30 mg biotin, 0.0275 mg vitamin B_12_, 10 mg riboflavin, 2.5 mg folic acid, 5 mg thiamin, 10,000 IU vitamin D_3_ (cholecalciferol), 125 mg vitamin E (tocopherol), 5 mg vitamin K_3_, 112.5 mg nicotinic acid, 37.5 pantothenic acid, 3.75 mg pyridoxine, 10 mg copper, 3 mg iodine, 50 mg iron, 60 mg manganese, 50 mg zinc, 0.75 mg selenium

*Metabolic Energy was calculated by formulation purposed by Carpenter and Clegs (1956)

### Determination of laying performance parameters

The quails’ body weights were recorded at the start and conclusion of the experiment using a highly accurate digital scale (± 0.01 g) to assess variations in their mass which will be established as body weight gain. The amount of feed consumed per cage was determined by measuring the difference between the initial feed quantity provided and the remaining feed after each period. The daily feed intake was calculated by dividing the total feed intake by the number of quails and the duration of the experiment and expressed as g per bird per day.

Throughout the experiment, eggs were gathered daily at a consistent time (16:00) and recorded to determine egg production, which was expressed as a percentage of the total quails per cage. Separately, damaged or cracked eggs were tallied, and their ratio to overall egg production was computed. In the last three days of the study, individual eggs were weighed on a precision scale (± 0.01 g) to evaluate egg weight. The egg production percentage was then multiplied by the mean egg weight to calculate the total egg mass. The feed conversion ratio (FCR) was calculated by dividing feed intake by egg mass, following the approach outlined in İflazoğlu Mutlu et al. (2021).

### Determination of egg quality traits

After the end of the experiment, six eggs from each cage (*n* = 30 eggs per group) were individually weighed and then cracked open on a clean, smooth glass surface. The eggshells were carefully separated, air-dried at room temperature for three days, and then weighed to determine their weight as a proportion of the total egg weight. Eggshell thickness was measured using a digital calliper (Tresna®, Guilin, China) with an accuracy of 0.01 mm. Subsequently, the eggshells were ashed at 550 °C in a muffle furnace (Nüve MF 106) to quantify their crude ash content (İflazoğlu Mutlu et al. 2021).

Internal quality parameters of the cracked eggs were assessed by evaluating both the albumen and yolk characteristics. Albumen and yolk heights were measured with a height gauge, while the length and width were determined using a digital calliper (Tresna®, Guilin, China). The yolk index, albumen index, and Haugh unit were calculated following the equations provided by Kamel et al (2021) and İflazoğlu Mutlu et al. (2021). Yolk colour was evaluated using a Roche yolk colour fan. Two eggs from each cage (10 eggs per group) were pooled without separating the yolk from the albumen and then dried in a hot air oven at 50 °C until a constant weight was achieved. The dried eggs were ground, and crude protein and ether extract content were analysed according to the methods described by AOAC (2006).

Additionally, two eggs were collected from each cage (10 eggs per group) to determine the carotenoid and cholesterol content of the yolks. The total carotenoid concentration was measured using the procedure outlined by Bidura et al. (2020) and expressed as µg/100 g of egg yolk. To assess total cholesterol, 0.1 g of hard-boiled yolk was homogenised with 5 mL of isopropyl alcohol and vortexed thoroughly. The resulting mixture was centrifuged at 3000 rpm for 10 min, and the supernatant was analysed using an autoanalyser equipped with a total cholesterol test kit. The cholesterol content was expressed in milligrams per gram of yolk.

### Determination of the antioxidant and lipid peroxidation status of egg yolk

Two eggs from each cage (*n* = 10 eggs per group) were collected to perform the analyses to determine the antioxidant capacity and lipid peroxidation levels of the egg yolks. For this purpose, quantities of malondialdehyde (MDA) and DPPH were determined. Each assay was conducted in triplicate to establish a mean value. The thiobarbituric acid reactive substances (TBARS) test was conducted according to those described by Zeb and Ullah (2016). One gram of egg yolk was extracted with 5 mL glacial acetic acid containing 0.01% Butylated hydroxytoluene (BHT) in an orbital shaker for an hour at room temperature. The supernatant was obtained by centrifuging the extract and 1 mL of the extract was combined with 1 mL of 4 mM Thiobarbituric acid (TBA). The mixture was heated at 95 °C for an hour and then placed on ice to halt the reaction. The absorbance of the solution was measured at 532 nm, which was within the linear range of the malondialdehyde (MDA) standard. The TBARS value was reported in terms of μM MDA per gram of yolk.

Additionally, the radical scavenging activity of the yolk extracts was evaluated using a modified technique of DPPH radicals (Sacchetti et al. 2005). Two grams of yolk samples were homogenised in 95% methanol, sonicated, and filtered. The resulting extracts were mixed with a DPPH solution and left to react for 30 min before reading the absorbance at 517 nm wavelength in a spectrophotometer (Perkin Elmer precisely UV/VIS Spectrometer). The DPPH value was established according to the formula reported by Sacchetti et al. (2005).

### Statistical analysis

The collected data were analysed using one-way analysis of variance (ANOVA) in a completely randomised design with SPSS software (version 26.0; SPSS Inc., Chicago, IL, USA) (SPSS 2018). For growth performance and egg production parameters, the mean values of each cage were considered as the experimental unit, whereas individual measurements were used as the experimental unit for the rest of the assessed traits. Tukey’s multiple comparison test was employed to identify significant differences between means, and the values of the tables were expressed as means ± standard error of the mean. Statistical significance was set at *P* < 0.05, while values of 0.05 ≤ *P* ≤ 0.10 were interpreted as indicative of a trend.

## Results

No mortalities or signs of illness were noted in any experimental group during the trial. As shown in Table 3, increasing levels of RCW inclusion had no significant effect (*P* > 0.05) on growth performance parameters, including final body weight, body weight gain, feed intake, and feed conversion ratio (FCR), as well as on egg production traits such as egg mass, egg production, and egg weight.

**Table 3:** Effects of red cabbage waste on performance and egg production traits of laying Japanese quails (*n* = 20)

Parameters	Red Cabbage Waste (%)				S.E.M	*P*-Value
	**0.0**	**0.5**	**1.0**	**2.0**		
Initial body weight	258.96	259.17	259.28	259.08	2.663	1.000
Final body weight	264.63	274.21	262.62	267.42	4.541	0.843
Body weight gain	5.68	15.05	3.35	8.34	4.251	0.814
Egg production	95.47	96.30	96.30	94.59	0.797	0.873
Egg weight	12.97	12.90	12.88	12.59	0.100	0.651
Egg mass	12.53	12.39	12.30	11.91	0.181	0.685
Feed intake	35.72	36.65	36.44	37.54	0.741	0.877
FCR	2.87	2.95	2.98	3.17	0.076	0.600

*S.E.M *Standard Error of the Mean, *FCR *Feed Conversion Ratio. Initial body weight, final body weight, and body weight gain are expressed as g. Feed intake is expressed as g/ day/quails. The feed conversion ratio is shown as g feed/ g egg. Egg production is expressed as a percentage. The egg mass is shown as g/day/quail

Table 4 summarises the internal egg quality traits. RCW inclusion did not significantly influence the yolk index, total cholesterol, or total carotenoid content (*P* > 0.05). However, distinct trends were observed for some of the internal quality traits. For instance, the Haugh unit and the albumin index were significantly higher in the 0.5% RCW group compared to the 2.0% RCW group, with the other groups displaying intermediate values. In contrast, the egg yolk colour index was significantly lower in all RCW-supplemented groups (*P* < 0.05) compared to the control.

**Table 4:** Effects of red cabbage waste on the internal egg quality (*n* = 120) of laying Japanese quails

Parameters	Red cabbage waste (%)				S.E.M	*P*-Value
	**0.0**	**0.5**	**1.0**	**2.0**		
Albumin Index	3.28^ab^	3.60^a^	3.47^ab^	3.06^b^	0.070	0.044
Yolk Index	49.93	49.24	51.45	50.86	0.637	0.649
Haugh Unit	91.20^ab^	93.97^a^	91.98^ab^	88.93^b^	0.572	0.023
Yolk Colour Score	11.29^a^	8.67^b^	8.63^b^	9.05^b^	4.508	0.000
Total Cholesterol	643.78	664.80	653.03	652.07	2.693	0.433
Total Carotenoids	26.62	25.14	21.44	31.79	0.134	0.636
Crude Protein	49.07^ab^	48.52^b^	48.74^b^	49.63^a^	0.173	0.010
Ether Extract	37.88^b^	39.28^a^	38.85^ab^	37.94^b^	0.166	0.002

*S.E.M *Standard Error of the Mean. Total cholesterol is expressed as mg/per gram of yolk. Total Carotenoids is shown as μg/100 g yolk. Crude Protein and ether extract is shown as a percentage of Dry Matter

^a^^, b^Means not sharing a common superscript letter within the same row are significantly different (*P* < 0.05)

In terms of the chemical composition of the yolk, the protein content was significantly reduced in the 0.5% and 1.0% RCW groups compared to the 2.0% RCW group, with the control group showing intermediate values. Conversely, the ether extract content of the yolk was significantly higher in quails fed the 0.5% RCW diet compared to both the control and 2.0% RCW groups, while the other groups displayed intermediate values.

The influence of incremental RCW supplementation on external egg quality parameters is presented in Table 5. No significant differences were noted in the percentage of damaged eggs between groups (*P* > 0.05), indicating consistent values across all diets. However, variations were observed in other external egg traits. Specifically, the eggshell ratio was significantly higher in the control group compared to the 1.0% RCW group, with the remaining groups displaying intermediate values. Similarly, eggshell thickness was significantly reduced in the 1.0% RCW group in comparison to the control group, while the other groups exhibited intermediate values. Regarding eggshell ash content, the 1.0% RCW group showed the lowest values, which were significantly different from both the control and 2.0% RCW groups.

**Table 5:** Effects of red cabbage waste on external egg quality (n = 120) in laying Japanese quail

Parameters	Red Cabbage Waste (%)				S.E.M	*P*-Value
	**0.0**	**0.5**	**1.0**	**2.0**		
Damaged egg	0.33	0.86	0.39	0.43	0.105	0.747
Eggshell ratio	8.56^a^	8.29^ab^	7.60^b^	8.18^ab^	0.113	0.016
Eggshell thickness	281.70^ab^	287.11^a^	253.37^b^	269.50^ab^	4.681	0.049
Eggshell ash	79.92^a^	79.50^ab^	76.04^c^	77.71^bc^	0.410	0.000

*S.E.M *Standard Error of the Mean. Damaged egg, Eggshell ratio and eggshell ash are shown as a percentage. Eggshell thickness is expressed as µm

^a^^−^^c ^means for not sharing a common superscript letter within the same row are significantly different (*P* < 0.05)

Finally, the results for the oxidative stability of egg yolks are presented in Table 6. There were significant differences in MDA and DPPH values across the treatment groups (*P* < 0.05). A linear increase in DPPH values was seen with the rise in RCW concentration in the diet. In contrast, the effect of RCW on MDA content was less consistent. The yolk MDA concentration was significantly lower in the 0.5% RCW group compared to the 1.0% RCW group, with the control and the other groups showing intermediate values.

**Table 6:** Effects of red cabbage waste on yolk (*n* = 40) antioxidant stability in laying Japanese quails

Parameters	Red cabbage waste (%)				S.E.M	*P*-Value
	**0.0**	**0.5**	**1.0**	**2.0**		
DPPH (% inhibition)	5.19^b^	7.54^ab^	6.52^ab^	8.30^a^	0.37	0.014
MDA (μM/g yolk)	1.27^ab^	1.11^b^	1.48^a^	1.17^ab^	0.04	0.004

S.E.M. Standard Error of the Mean. DPPH: 2,2 diphenyl-1-picrylhydrazyl. MDA: Malondialdehyde

^a^^, b^Means not sharing a common superscript letter within the same row are significantly different (*P* < 0.05)

## Discussion

### Quail development and egg production

Red cabbage is a nutrient-dense vegetable, which is rich in nutrients and polyphenols which makes it a valuable addition to the human diet that may provide favourable effects on its health (Ghareaghajlou et al. 2021), and a promising option to be used as a feed additive for animal nutrition (Nunes et al. 2019). In the current research, no significant differences were observed in growth performance or egg production parameters among the experimental group. Previous authors suggest that the improvement in performance parameters linked to byproduct diet could be more evident when the animal’s physiological state, background nutrition, or housing conditions are compromised (Sarmiento-García et al. 2024). Nevertheless, our findings are consistent with previous research demonstrating that polyphenol-rich by-products, such as red cabbage, are generally safe for poultry and do not adversely affect performance traits. For example, the inclusion of dried cabbage leaves in laying hens’ diets (Mustafa and Baurhoo 2018), red beet powder (Sarmiento-García et al. 2023a), dried cassava residue (Almeida et al. 2020), and dehydrated bocaiuva pulp (Nunes et al. 2019) in the diets of laying quails did not influence body weight gain, feed intake, egg production, egg weight, egg mass, or feed conversion ratio. In any case, the results of this study indicate that including RCW in the diet of laying quails is an effective way to reuse by-products without compromising development or egg production parameters, and provides a promising approach to reducing feed costs.

### Egg internal parameters

The Haugh unit and albumen index are widely recognised as essential parameters for assessing albumen quality and are indicative of egg freshness (Chen et al. 2021). In the current research, including 0.5% RCW in the diets of laying quails significantly enhanced both the albumen index and Haugh unit compared to 2.0% RCW. These findings align with previous research on polyphenol-rich by-products, which have shown similar enhancements. For instance, Chen et al. (2021) demonstrated that magnolol, a polyphenol from Magnolia, improved both the Haugh unit and albumen height, while Sharmin et al. (2021) reported that *Moringa oleifera* increased the albumen index in fresh eggs. Similarly, Ahmed et al. (2022) found that dried orange peel supplementation in Japanese quails boosted the Haugh unit and albumen index. The observed improvements may be linked to the antioxidant properties of polyphenols in RCW, which could reduce oxidative stress in laying birds, enhancing albumen quality. Polyphenols are known to promote better gut health and nutrient absorption, which may influence albumen synthesis. Conversely, the decline in these parameters with 2.0% RCW could be due to a threshold effect, where higher polyphenol concentrations interfere with nutrient absorption or metabolic processes, potentially leading to a negative impact on protein utilisation and egg quality. Further investigation into the dose-dependent effects of polyphenols on albumen formation and egg freshness is warranted to clarify these mechanisms.

Yolk colour plays a crucial role in consumer decisions (Sarmiento-García et al. 2023b). In the current research, including RCW in laying quail diets resulted in a significant reduction in egg yolk colour score. As described by Sosnówka-Czajka and Skomorucha (2021) yolk pigmentation is primarily influenced by the absorption and deposition of carotenoids in the egg yolk, which are absorbed from the intestine of poultry. Hence, the observed reduction in yolk colour in the present study could be attributed to the effects of red cabbage on carotenoid bioavailability. Previous research has shown that red cabbage may interfere with the bioaccessibility of carotenoids in co-digested foods by affecting their stability during digestion (Phan et al. 2019). Consequently, the lower yolk colour scores in quails-fed RCW diets may be due to a decrease in carotenoid absorption, limiting their deposition in the yolk.

This study found a decrease in egg crude protein levels in groups receiving 0.5% and 1.0% RCW, while the 2.0% RCW group had relatively higher levels. Although polyphenols are known to enhance nutrient absorption by improving gut health, they may also reduce protein digestion through enzyme inhibition at certain concentrations (Corrêa et al. 2019). Anthocyanins in RCW have been shown to modulate gut microbiota and inhibit digestive enzymes, which may explain the lower protein levels in eggs at 0.5% and 1.0% RCW (Mattioli et al. 2020). This suggests that specific concentrations of polyphenols can interfere with protein digestion by forming complexes with enzymes like proteases. Therefore, it is plausible that at lower concentrations (0.5% and 1.0%), the polyphenols in RCW interfered with protein digestion, leading to lower egg crude protein levels.

In contrast, our results showed that supplementation with 2.0% RCW decreased egg yolk fat content, while the 0.5% RCW group exhibited the highest fat content. This could be linked to the mobilization of body fat by anthocyanidins, which are known to enhance lipid metabolism by promoting fat breakdown and inhibiting fat accumulation (Corrêa et al. 2019), leading to less lipid deposition in the eggs. At lower RCW levels (0.5%), insufficient polyphenol activity may not have stimulated fat mobilization, resulting in higher fat deposition in the yolk. Similarly, Dedousi et al. (2022) observed that anthocyanin-rich dried olive pulp increased egg fat content at moderate inclusion rates but reduced it at higher levels.

These findings suggest a complex interaction between RCW supplementation, egg protein content, and yolk fat deposition, with polyphenol concentration playing a pivotal role in modulating these parameters. While higher concentrations of RCW (2.0%) appear to enhance protein deposition and reduce fat content, lower concentrations (0.5%) may favour fat accumulation in the yolk. Further studies are necessary to elucidate the mechanisms by which different polyphenol concentrations impact nutrient metabolism in laying quails.

### Egg external parameters

In this study, quail-fed diets containing 0.5% RCW exhibited higher eggshell thickness, compared to birds receiving 1.0%. Moreover, 0.5% RCW has a numerically higher eggshell thickness value compared to those received RCW at 2.0%. This could suggest a dose-dependent effect of RCW on eggshell quality, with moderate inclusion levels (0.5%) enhancing thickness, while higher levels (1.0% and 2.0%) may exert a detrimental effect. Although most of the studies conducted on using polyphenol-rich by-products as feed additives improved eggshell quality, dietary inclusion of tea polyphenols showed reduced eggshell thickness in laying hens. This could be attributed to excessively high levels of polyphenols interfering with calcium absorption and promoting calcium loss in the animals’ metabolism (Abd El‐Hack et al. 2023).

Additionally, the eggshell ash ratio, indicative of the mineral content and overall structural integrity of the eggshell, was significantly reduced in groups with increasing amounts of RCW. The observed reduction in mineral content aligns with the theory that polyphenols, abundant in RCW, can interfere with mineral absorption. Similar findings were reported by Mustafa and Baurhoo (2018), where the eggshell ratio was reduced by the inclusion of 8% dried cabbage leaves in the diets of laying hens. Moreover, Nunes et al. (2019) found that including polyphenol-rich dehydrated bocaiúva pulp in the diet of laying quails resulted in a linear decrease in eggshell thickness. Contrarily, research on laying hens supplemented with anthocyanin-rich purple corn extract demonstrated improvements in eggshell thickness (Li et al. 2023). These discrepancies suggest that the effects of polyphenols on eggshell quality may vary depending on the specific type of polyphenol and its interaction with other dietary components. While the exact mechanisms by which polyphenols influence calcium metabolism remain unclear, anthocyanins have been proposed to enhance calcium absorption by modulating the intestinal environment and improving liver function (Li et al. 2023). However, it is also well-documented that certain polyphenols can chelate minerals, which may reduce their bioavailability and interfere with calcium absorption (Bohn 2014). This duality highlights the complex role of polyphenols in mineral metabolism, suggesting that their impact on eggshell quality is context-dependent, and potentially influenced by polyphenol type, concentration, and the overall dietary matrix.

### Antioxidant capacity

Bioactive compounds, particularly polyphenols, are known to be the primary contributors to the antioxidant capacity of plants (Sarmiento-García et al. 2023a). Red cabbage, being rich in polyphenols—especially anthocyanins, which impart its characteristic purple-red colour—has demonstrated significant antioxidant potential (Ghareaghajlou et al. 2021). In the current study, dietary inclusion of RCW resulted in a linear increase in DPPH inhibition of the yolk as the RCW dose increased, indicating enhanced antioxidant activity. This finding aligns with previous research showing improved antioxidant capacity by including polyphenol-rich ingredients such as dried orange pulp (Hussein et al. 2023) and purple corn extract (Li et al. 2023) in laying hen diets.

However, despite the increase in antioxidant capacity, measured by DPPH inhibition, the malondialdehyde (MDA) levels did not decrease in a similarly linear trend. The 1.0% RCW group exhibited the highest MDA levels, while the 0.5% group had the lowest MDA value, indicating an unexpected pattern. The expected inverse relationship between antioxidant activity and lipid peroxidation (as measured by MDA) was not previously described, which raises important questions about the dynamics of polyphenols under different dietary conditions. One potential explanation for these findings is the dual role that polyphenols can play depending on their concentration and environmental factors. Polyphenols are recognised for their ability to enhance gut health and nutrient absorption, which may, in turn, positively influence albumen synthesis (Abd El-Hack et al. 2023). This pro-oxidant effect could contribute to increased lipid oxidation, as seen in the 1.0% RCW group. Polyphenols can interact with metal ions and other reactive species, potentially promoting oxidative stress rather than mitigating it. This phenomenon has been observed in other studies as well; for example, Sarmiento-García et al. (2023a) found that increasing concentrations of red beet powder, rich in polyphenolic compounds, initially raised MDA levels in quails before higher doses eventually reduced lipid oxidation.

In this context, the results of our study suggest that the 1.0% RCW supplementation may have reached a concentration where pro-oxidant effects began to manifest, contributing to increased MDA levels. Hence, the relationship between polyphenol supplementation, antioxidant activity, and lipid peroxidation in egg yolks is complex and likely dependent on multiple factors, including polyphenol concentration, structure, and interaction with other dietary components (Abd El‐Hack et al. 2023). So, further research is needed to elucidate the precise mechanisms behind these effects and to optimise polyphenol use in poultry diets.

## Conclusion

This study shows that incorporating RCW in the diets of laying Japanese quails does not negatively impact their overall performance. Notably, supplementation with 0.5% RCW showed greater benefits for internal egg quality, particularly enhancing the albumen index and Haugh unit, compared to higher doses (2.0%). Additionally, the 0.5% RCW level could show an adequate antioxidant capacity, as indicated by reduced lipid peroxidation (MDA concentration) compared to higher doses (> 0.5%).

Including RCW in quail diets presents a sustainable strategy for improving egg quality while utilising food waste. This study highlights the optimal inclusion rate of 0.5% RCW, which enhances egg internal quality without compromising performance or causing oxidative stress. Further studies are recommended to fine-tune polyphenol concentrations and fully unlock the potential of RCW as a sustainable feed additive.

## Data Availability

The researchers affirmed that all the information and resources utilized in this analysis adhere to the established guidelines of the field and can be easily accessed upon request.
